# Supplementary material for: Sequential involvements of the perirhinal cortex and hippocampus in the recall of item-location associative memory in macaques
Source: PLoS Biol. 2023 Jun 8;21(6):e3002145. doi: 10.1371/journal.pbio.3002145 (PMC10284415; doi:10.1371/journal.pbio.3002145)
Supplement: S1 Table — Numbers of neurons showing item-cue effect during the item-cue period (P < 0.01, one-way ANOVA) and those showing the item-cue, context, and target effects during the choice-fixation period (P < 0.01, three-way ANOVA). “Item-cue” indicates an item effect during the item-cue period or choice-fixation period. “Context” indicates a context effect during the choice-fixation period. “Target” indicates a target location effect during the choice-fixation period. Source data are available in S1 Data. (DOCX) [file pbio.3002145.s001.docx]

|  |  | ***Item-cue period*** | ***Choice-fixation period*** | | |
| --- | --- | --- | --- | --- | --- |
|  | **Recorded** | **Item-cue** | **Item-cue** | **Context** | **Target** |
| ***Total*** |  |  |  |  |  |
| **TE** | 168 | 47 | 2 | 3 | 12 |
| **PRC** | 319 | 84 | 2 | 7 | 19 |
| **PHC** | 232 | 22 | 2 | 1 | 25 |
| **HPC** | 456 | 136 | 8 | 14 | 55 |
| ***Monkey B*** |  |  |  |  |  |
| **TE** | 98 | 36 | 2 | 1 | 8 |
| **PRC** | 168 | 29 | 1 | 2 | 14 |
| **PHC** | 70 | 7 | 1 | 0 | 7 |
| **HPC** | 247 | 66 | 5 | 7 | 38 |
| ***Monkey C*** |  |  |  |  |  |
| **TE** | 70 | 11 | 0 | 2 | 4 |
| **PRC** | 151 | 55 | 1 | 5 | 5 |
| **PHC** | 162 | 15 | 1 | 1 | 18 |
| **HPC** | 209 | 70 | 3 | 7 | 17 |
