# Supplementary figures and images for: Sequential involvements of the perirhinal cortex and hippocampus in the recall of item-location associative memory in macaques
Source: PLoS Biol. 2023 Jun 8;21(6):e3002145. doi: 10.1371/journal.pbio.3002145 (PMC10284415; doi:10.1371/journal.pbio.3002145)

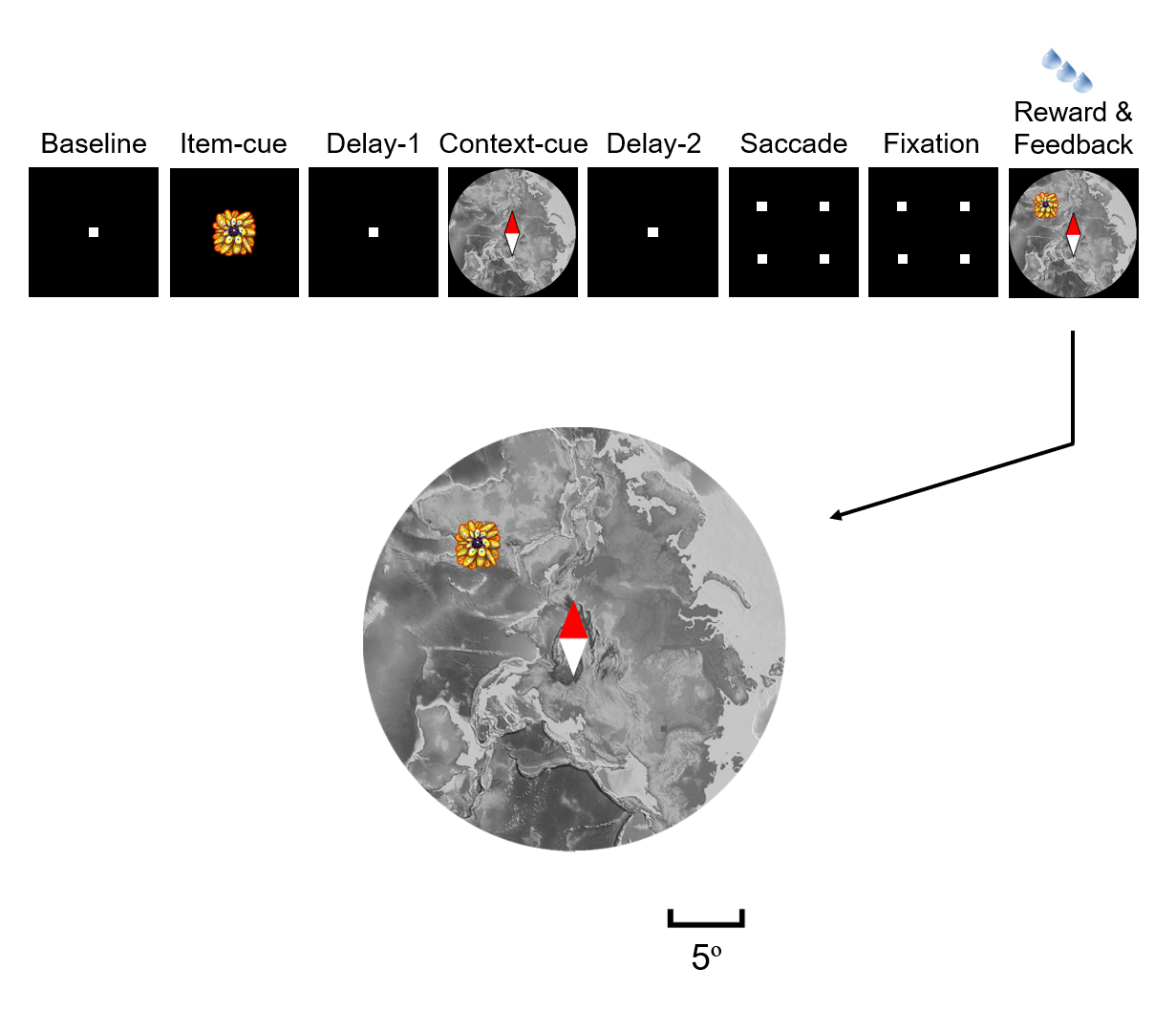

Supplement: S1 Fig — The visual feedback image of 1 example trial was magnified. Scale bar for feedback image, 5° visual angle. The background map image was made based on an image (EMU 13) from public domain, “USGS” (https://www.usgs.gov/media/images/emu-13). (TIF) [file pbio.3002145.s002.tif]

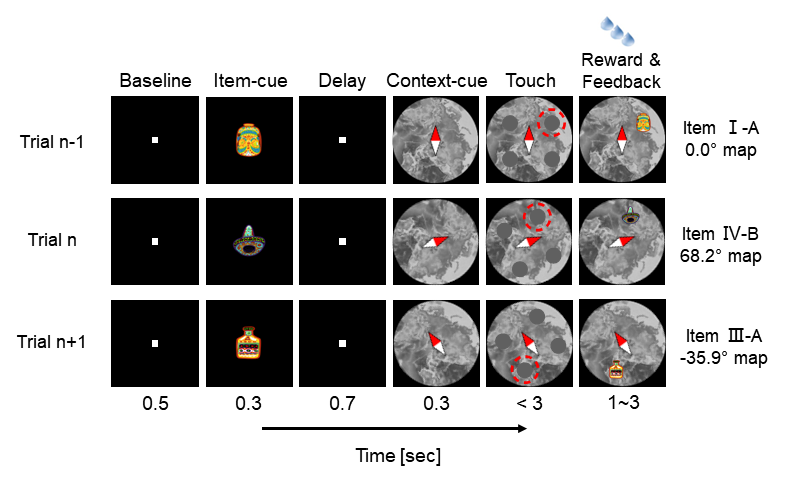

Supplement: S2 Fig — An item-cue and a context-cue were sequentially presented in each trial. The item-cue was chosen randomly from the 8 visual items, and the context-cue was presented with a randomly chosen orientation from −90° to 90° in a 0.1° step. Monkeys had to make a choice by touching the target location (red dashed circle) according to the 2 cues. A successful trial was rewarded with juice paired with feedback showing the associated location of the item-cue on the context-cue. Relative sizes of the stimuli were magnified for display purposes. The background map image was made based on an image (EMU 13) from public domain, “USGS” (https://www.usgs.gov/media/images/emu-13). (TIF) [file pbio.3002145.s003.TIF]

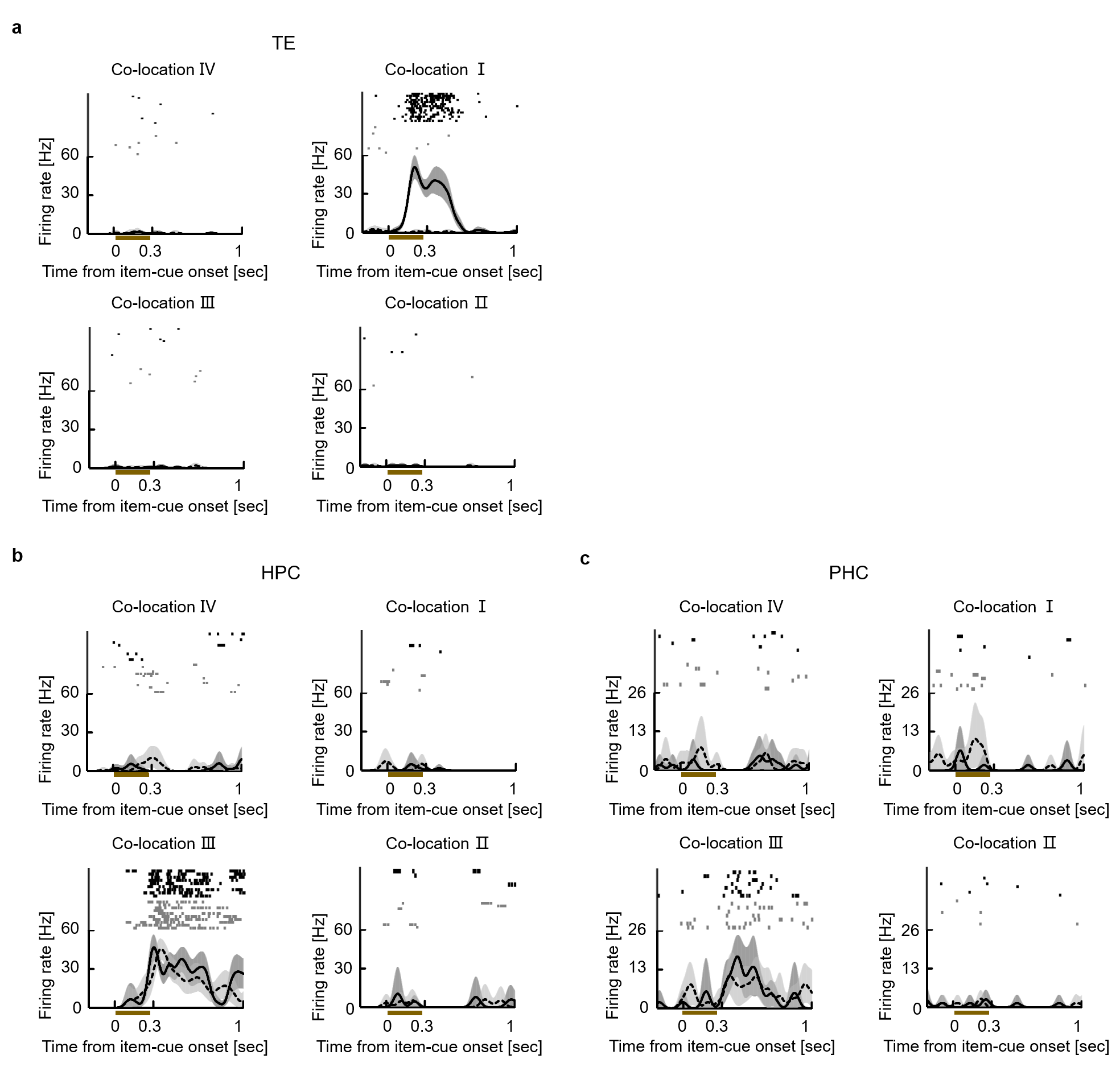

Supplement: S3 Fig — Following a similar format as Fig 3A. Solid lines and dashed lines indicate SDFs in trials with item-cues from the stimulus sets A and B, respectively. Dark and light gray shading, 90% confidence interval of 10,000 bootstraps for the stimulus sets A and B, respectively. Black and gray dots, raster plots for the stimulus sets A and B, respectively. Brown bar, presentation of the item-cue. (a) An example neuron from TE. The neuron showed the item-cue selective activities but not the co-location effect. P < 0.0001, F (7,115) = 153.41, one-way ANOVA. Co-location index r = 0.16, Pearson correlation; P = 0.87, two-tailed permutation test. (b) An example neuron from the HPC. The neuron showed the co-location effect on the item-cue selective activities. P < 0.0001, F (7,70) = 45.09, one-way ANOVA. Co-location index r = 0.99, Pearson correlation; P = 0.0002, two-tailed permutation test. (c) An example neuron from the PHC. The neuron showed the co-location effect on the item-cue selective activities. P < 0.0001, F (7,57) = 15.74, one-way ANOVA. Co-location index r = 0.99, Pearson correlation; P = 0.0004, two-tailed permutation test. Source data are available in S1 Data. (TIF) [file pbio.3002145.s004.tif]

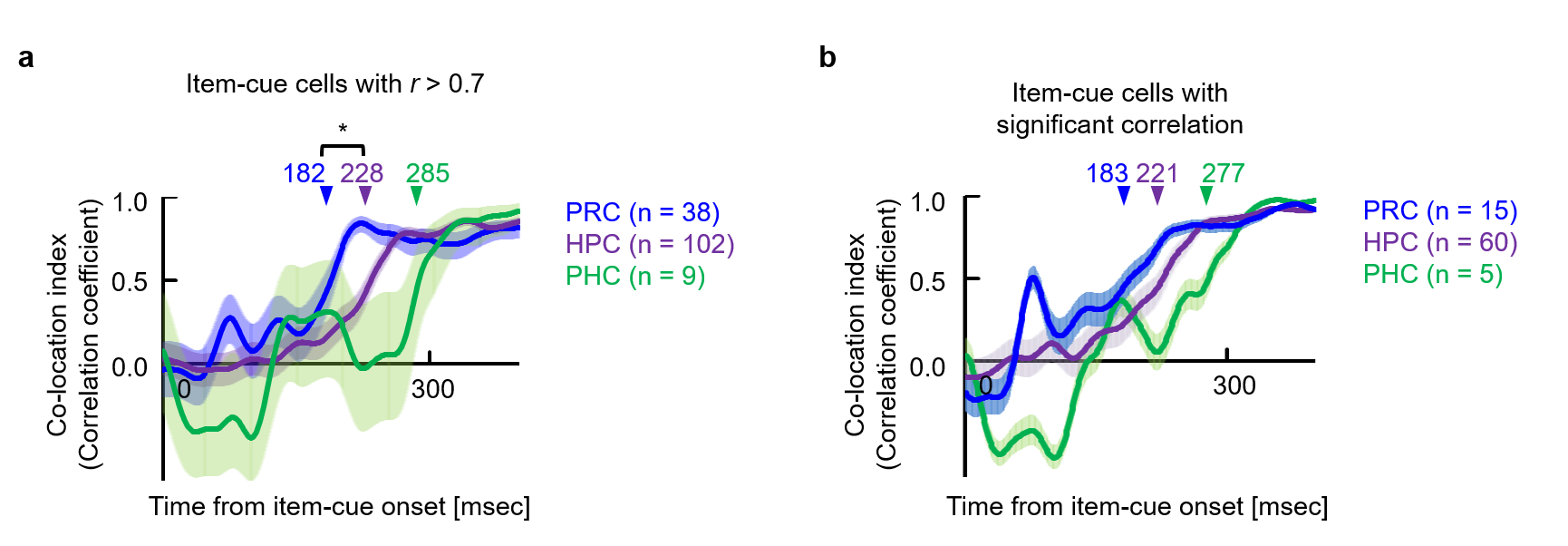

Supplement: S4 Fig — (a) For the item-cue selective neurons with high co-location indices (r > 0.7) in PRC (blue, n = 38), HPC (purple, n = 102), and PHC (green, n = 9). The formats are the same as those in Fig 4C. The co-location index increased earlier in the PRC than in the HPC (P = 0.0114*, two-tailed permutation test). (b) For item-cue selective neurons with significant co-location effect (P < 0.05, two-tailed permutation test) in PRC (blue, n = 15), HPC (purple, n = 60), and PHC (green, n = 5). The formats are the same as those in Fig 4C. Source data are available in S1 Data. (TIF) [file pbio.3002145.s005.tif]

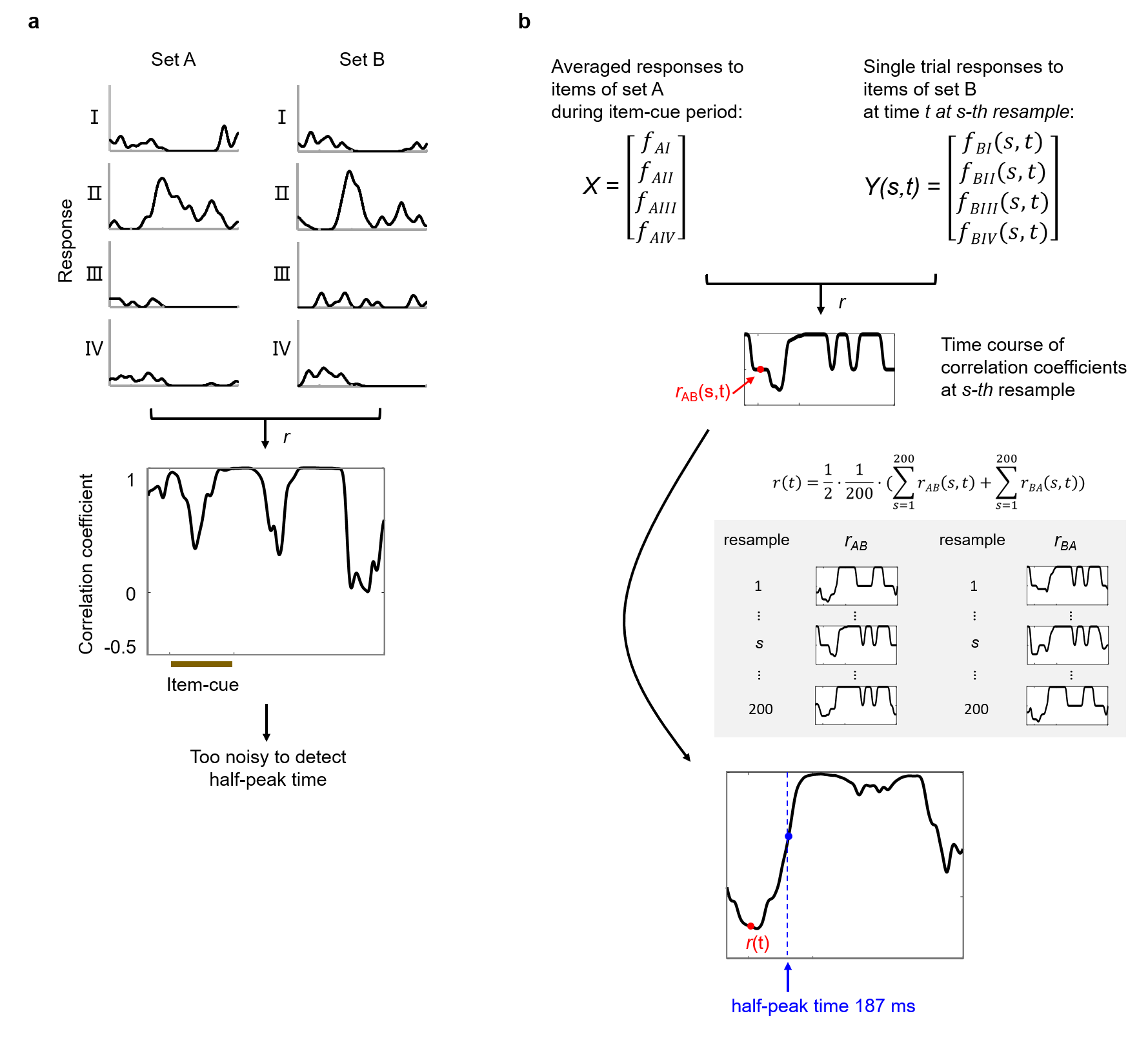

Supplement: S5 Fig — (a) PSTHs and time course of the co-location index of an example item-cue selective neuron with a high colocation index (0.97). The neuron showed a large value of the co-location index even before the item-cue presentation. This type of noise tends to occur because of the small degree of freedom in the correlation coefficient. (b) To eliminate the inevitable noise component, we employed the trial resample and average method within an individual neuron. Template vector X, average responses across trials to each of the 4 items of 1 set during the 60- to 1,000-ms period from item-cue onset. Either set A or set B was chosen as the template vector alternatively. Temporally variable vector Y(s,t), responses of 1 trial at s-th resample to each of the 4 items of the other set at each time “t”; t, the center of 100-ms time-bin moving by 1 ms during the item-cue period. For each set, vector Y was generated 200 times by resampling trials. The dimensions of the 2 vectors were both 4. rAB(s,t), the correlation coefficient between X and Y at s-th resample at time t wherein the template vector was generated from set A and the temporally variable vector was generated from set B, s = 1 to 200. r(t), the average correlation coefficient at time t. The half-peak time of each neuron was calculated from the time course of the average correlation coefficient. (TIF) [file pbio.3002145.s006.tif]

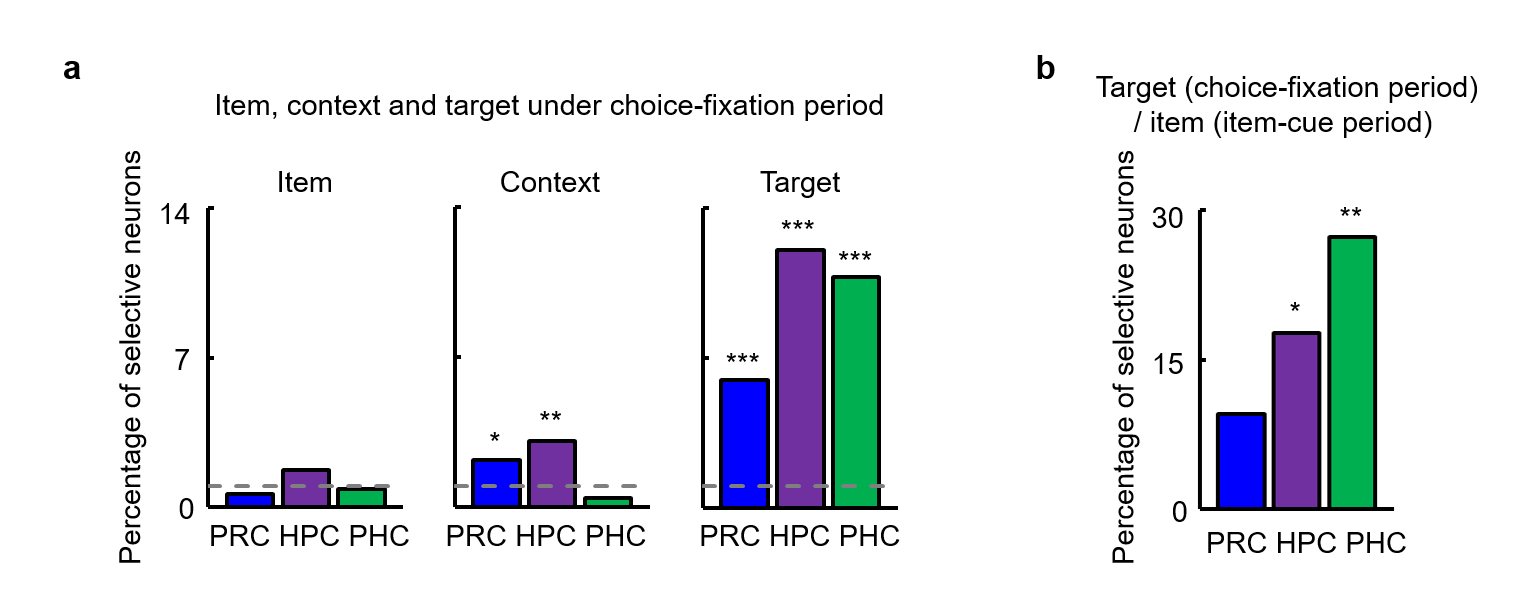

Supplement: S6 Fig — (a) Percentages of item-cue, context-cue, and target-selective neurons out of the recorded neurons during the choice-fixation period. PRC, n = 319; HPC, n = 456; PHC, n = 232. Dashed line, 1.0% chance level. Asterisks indicate results of one-tailed binomial test (probability of null hypothesis = 1.0%): P = 0.0432* for context-cue selective neurons in PRC; P = 0.0003** for context-cue selective neurons in the HPC; P < 0.0001*** for target-selective neurons in each area. (b) Percentage of target-selective neurons during choice-fixation period out of item-cue selective neurons (PRC, n = 84; HPC, n = 136; PHC, n = 22). Asterisks indicate results of a χ-square test: P = 0.0170*, χ2 = 5.7, d.f. = 1; P = 0.0087**, χ2 = 6.88, d.f. = 1. Source data are available in S1 Data. (TIF) [file pbio.3002145.s007.tif]

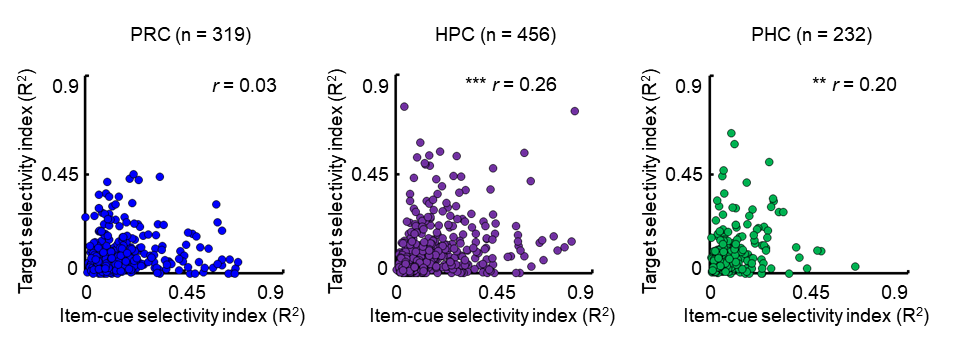

Supplement: S7 Fig — Item-cue selectivity index, the R2 value of item effect from one-way ANOVA test during the item-cue period. Target selectivity index, the R2 value of target location effect from three-way ANOVA test during the choice-fixation period. Each dot indicates 1 neuron. The significant correlations between the item-cue selectivity index and target selectivity index were found in the HPC (P < 0.0001***, two-tailed permutation test) and PHC (P = 0.0084**). Source data are available in S1 Data. (TIF) [file pbio.3002145.s008.TIF]

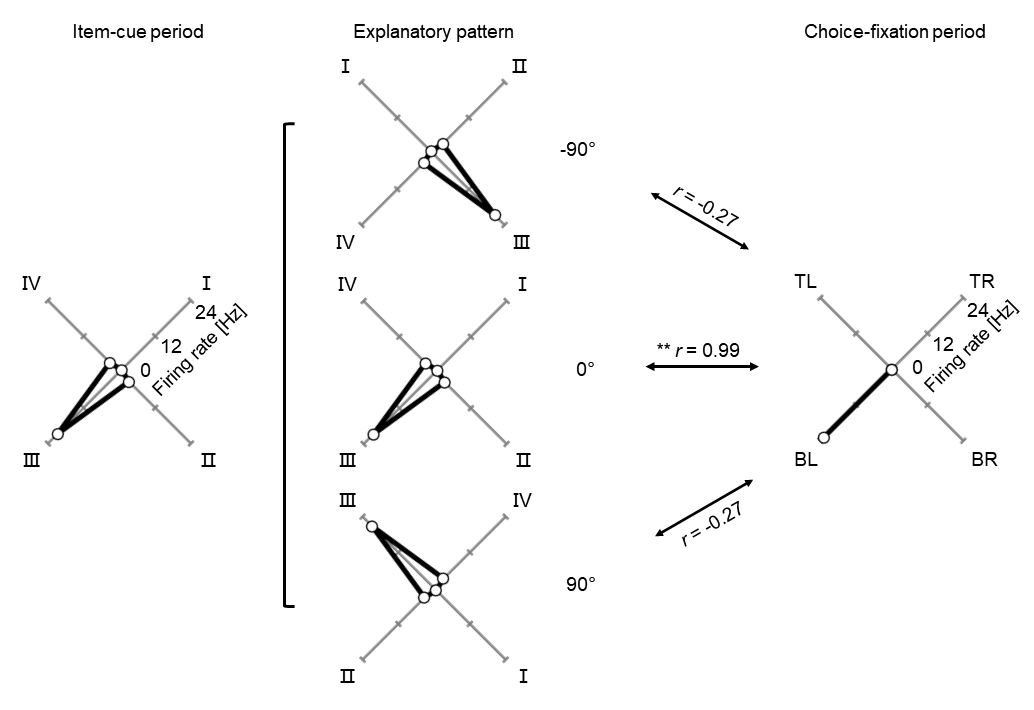

Supplement: S8 Fig — Explanatory pattern, the co-locations were assumedly positioned relative to a −90°, 0°, or 90° context-cue. r, the similarity between response patterns to the co-locations during the item-cue period and those to the target locations during the choice-fixation period. I-IV, co-location I-IV. TR, top-right; BR, bottom-right; BL, bottom-left; TL, top-left. Asterisks indicate the results of a two-tailed permutation test: P = 0.0074**. Source data are available in S1 Data. (TIF) [file pbio.3002145.s009.TIF]

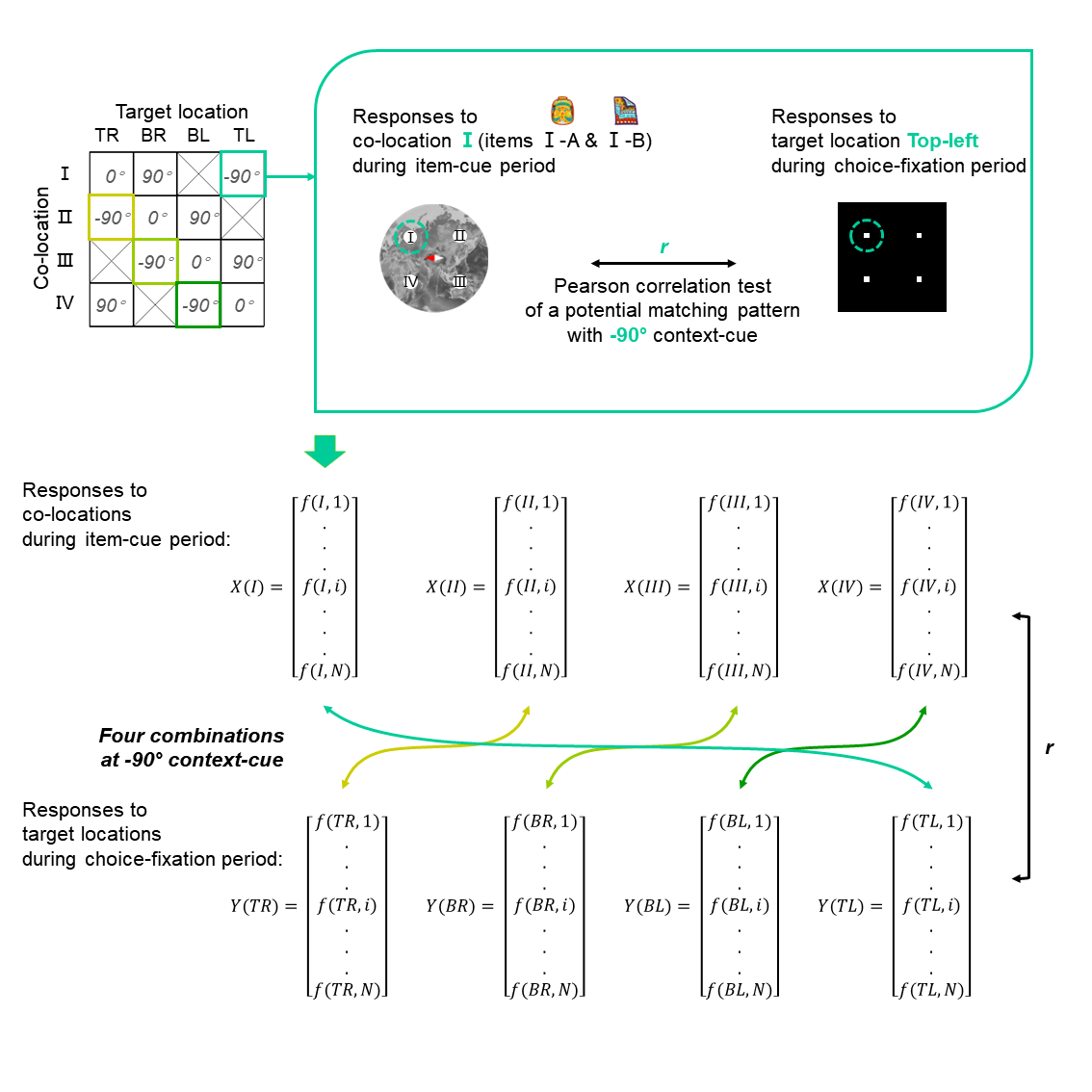

Supplement: S9 Fig — X(co-location), N-dimensional population vector for each co-location. f(co-location, i), the average firing rate of the i-th neuron for the co-location during the item-cue period. N, number of recorded neurons in the area. Y(target), population vector for each target location. f(target, i), the average firing rate of the i-th neuron for the target location during the choice-fixation period. r, Pearson correlation coefficient between X(co-location) and Y(target). −90°, 0°, or 90°, explanatory pattern, the co-locations were assumedly positioned relative to a −90°, 0°, or 90° context-cue. The background map image was made based on an image (EMU 13) from public domain, “USGS” (https://www.usgs.gov/media/images/emu-13). (TIF) [file pbio.3002145.s010.TIF]

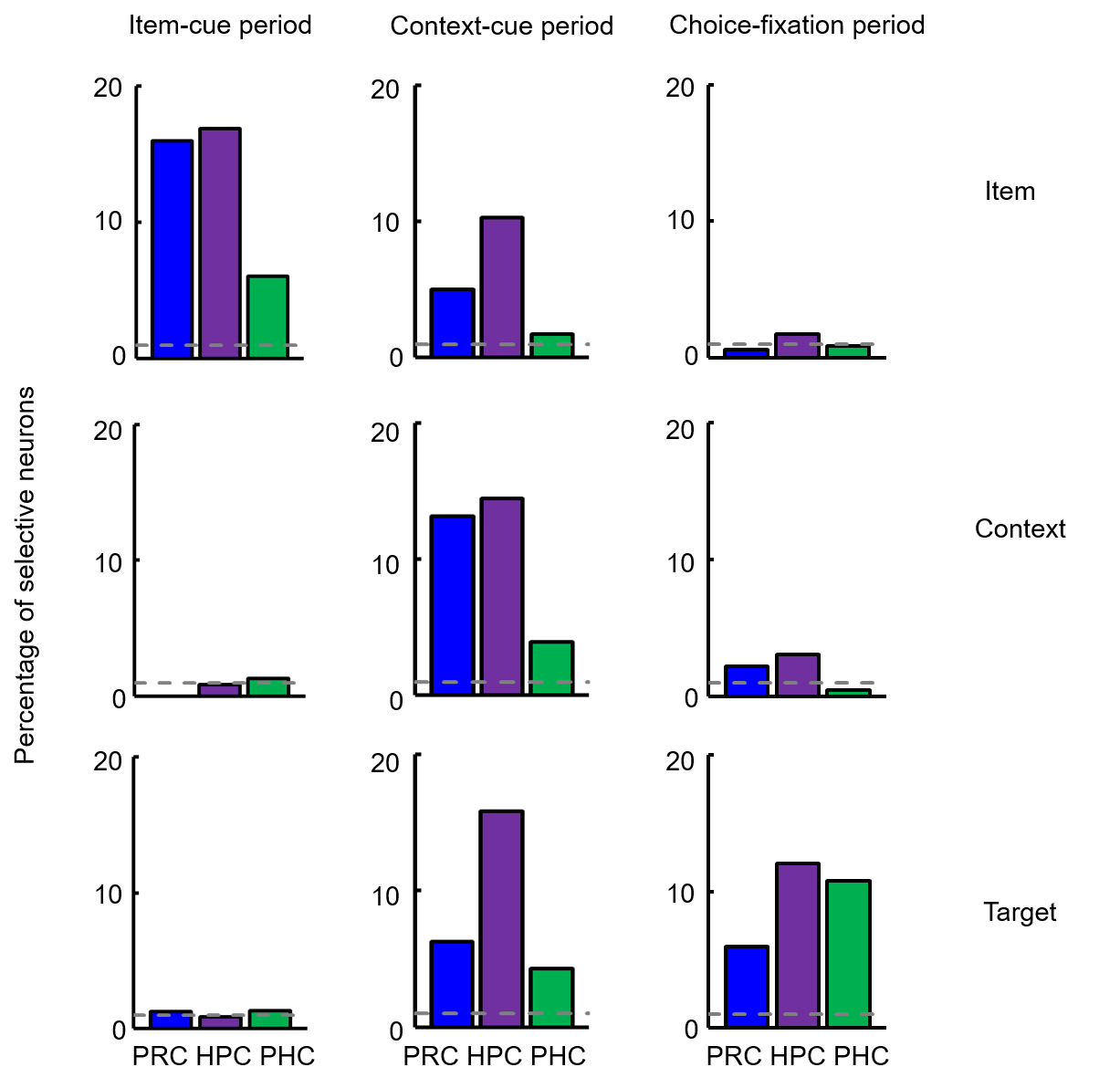

Supplement: S10 Fig — Percentages of item-cue (top row), context-cue (middle row), and target location (bottom row) selective neurons out of the recorded neurons during 3 periods. Left column, item-cue period, 0–1,000 ms from item-cue onset, three-way ANOVA (P < 0.01, Bonferroni correction for eight-analysis time windows). Middle column, context-cue period, 0–1,000 ms from context-cue onset, three-way ANOVA (P < 0.01, Bonferroni correction for eight-analysis time windows). Right column, choice-fixation period, 0–200 ms from choice-fixation onset, three-way ANOVA (P < 0.01). “Item,” “context,” and “target” effects for each neuron were evaluated across all correct trials with −90°, 0°, and 90° context-cues. PRC, n = 319; HPC, n = 456; PHC, n = 232. Dashed line, 1.0% chance level. Source data are available in S1 Data. (TIF) [file pbio.3002145.s011.tif]
