# Supplementary material for: Sequential involvements of the perirhinal cortex and hippocampus in the recall of item-location associative memory in macaques
Source: PLoS Biol. 2023 Jun 8;21(6):e3002145. doi: 10.1371/journal.pbio.3002145 (PMC10284415; doi:10.1371/journal.pbio.3002145)
Supplement: Attachment — Submitted filename: Response_Yang_Naya.pdf [file pbio.3002145.s013.pdf]

Responses to Reviewer #1:

Thank you for the recognition of our work and the important suggestions and comments on the description of our manuscript. We have addressed all the comments listed point-by-point and have revised our manuscript accordingly.

Specific comments: major

Comment 1: In the field of learning and memory, there are many psychologists and neuroscientists, including both neurophysiologists and neuropsychologists, who simply do not believe that macaques (or other nonhuman animals) possess declarative memory. For the authors to make the assumption that macaques have declarative memory, and that it works just like human declarative memory, is overreaching. Books devoted to the exploration of the topic include (The Gap, by Suddendorf; The Evolution of Memory Systems, by Murray, Wise and Graham). While the findings of the present study are novel, I would urge the authors to present them in the context of associative learning. It is certainly fair enough to make the case in the Discussion that the present findings align with aspects of semantic memory in humans, but even that requires some explanation for the emphasis on perirhinal cortex and not area TE. It is evident that semantic memory in humans centers not on perirhinal cortex, but on a more lateral portion of temporal cortex that represents features at a categorical level (L Tyler et al., J Cogn Neurosci 2013; DA Levy et al., PNAS 2004; R Insausti et al., PNAS 2013). If this is the case, how do the authors explain the lack of area TE involvement in their semantic retrieval?

Please reframe the results in the context of associative learning, as opposed to the framework of human declarative memory.

Response: Thank you for raising this issue. We used the term, “semantic-like” in the title of the original manuscript because we were also concerned that “there are many psychologists and neuroscientists who do not believe that macaques (or other nonhuman animals) possess declarative memory.” According to the reviewer, we primarily used “item-location associative memory” to refer to the long-term memory that the present ILA task required, while we further specified the item-location associative memory by adding the term “semantic-like” in comparison with “episodic-like” (Page 1, Line 2; Page 2, Line 33 - Page 3, Line 35; Page 5, Line 78 - Page 6, Line 81; Page 6, Lines 99-100; Page 15, Lines 265-268; Page 18, Lines 323-326; Page 19, Lines 339-341, 351-355). We designed our neurobiological study using animal experiments to contribute to understanding one important aspect of semantic memory [e.g., allocentric and self-referenced (Buzsáki, McKenzie and Davachi, 2022)] which might be shared across species, especially between human and non-human primates, although it may not perfectly match to whole aspects of “semantic memory” referred to as “cultural knowledge” for humans (Murray, Wise and Graham, 2017).

Many previous studies have examined brain regions storing semantic memory using objects, names, and/or categories and found that semantic memory is mainly substantiated by the lateral part of the temporal lobe, which processes objects and object-related information (Insausti et al., 2013; Levy, Bayley and Squire, 2004; Tyler

et al., 2013). In contrast, this study required subjects to remember spatial information rather than objects. This unwanted recall of objects in the ILA task may explain the lack of area TE involvement in “semantic" retrieval. We have described this potential reason in the revised manuscript (Page 16, Lines 275-281).

Comment 2: It is somewhat surprising that area TE did not show co-location effects, which are essentially item-item associations, perhaps mediated via the map. The authors discuss this briefly in the Results section, explaining that TE is important for perception, not memory (lines 152-154). To this referee, however, the item-item paired associate learning (as exemplified by pair-coding effects) and the co-location effects are both examples of memory retrieval. There should be some discussion of the present results relative to the results of earlier work on item-item paired associated learning (e.g. S Higuchi and Miyashita, PNAS, 1996). Specifically, if area TE shows correlates of item-item retrieval in the paired-associates task, why are there no significant co-location effects in TE in the present study? This should be addressed in the Discussion.

Response: Thank you for bringing this point to our attention. We consider that there were no significant co-location effects in the TE for the following reasons. First, the correlated responses to co-locating items in the present ILA task essentially depend on item-location association rather than the direct item-item association shown in the item-item paired association paradigm as shown in the original manuscript. Second,

the ILA task required subjects to remember location information from the item-cue, but not the item information. This task demand may direct the retrieval signal to transmit forward from the PRC to HPC but not backward from the PRC to TE. Considering that the item-item association effect in TE depends on the feedback signal from the PRC in the paired association paradigm (Higuchi and Miyashita, 1996; Naya, Yoshida and Miyashita, 2001), the absence of the feedback signal from the PRC would explain the present result (no significant co-location effects in TE in the ILA task). We have discussed this notion in the revised manuscript according to your suggestion (Page 16, Lines 270-281).

Comment 3: The nature of the visual feedback is important for understanding how the monkeys learned the ILA task. The Reward and feedback column in Figure 1C is not large enough to see the detail. In the feedback period, is the item from the item-cue period shown in the correct location of the map? If not, what is given for feedback, beyond the reward itself? If there is visual feedback, please show a larger image of feedback column (either here or in Supplemental methods) and describe in the Methods. If there is no visual feedback beyond presentation of the map at the same orientation as in context-cue period, please state this is the case. What is the feedback after an incorrect response (error)?

Response: Thank you for raising this concern. Visual feedback is the item-cue overlap on the correct location of the context-cue. An incorrect response leads to an inter-trial

interval without feedback. We added an example of visual feedback with a larger size in Fig S1 and described them in the Methods section (Page 22, Line 395-399).

Specific comments: minor

Comment 1: Line 73: the authors say the monkeys needed to store the common relationship between the items and the map image. Strictly speaking, this is not the case. Monkeys can learn hundreds of specific visuomotor conditional associations - independently - even though one would have thought they learned a more general rule. The fact that the study did not use rotations beyond 90 degrees during the recording sessions suggest the monkeys had a hard time learning this task, and never quite grasped the semantic aspects of it. I suggest replacing needed to store with were encouraged to store or a similar phrase.

Response: Thank you for raising this concern. We have revised the manuscript accordingly (Page 5, Line 76).

Comment 2: Lines 79-80: the phrase positioning their gaze is awkward. It would be better to simply say the monkeys made a saccade to the location corresponding to the correct quadrant on the map or similar.

Response: Thank you for highlighting this point. We simplified that phrase as "made a saccade to the location assigned to the item-cue relative to the context-cue" in the revised manuscript (Page 6, Line 83-84).

Comment 3: Lines 109-110: the phrasing the monkeys reported a target location which corresponded to the relative location of the co-location of the item cue is awkward. As I understand it, the monkeys simply made a saccade to the target location corresponding to the item location on the map, taking into account the orientation of the map on each trial. In performing the task, co-location is not relevant; the monkeys may know that two items correspond to each quadrant, but that may not be important to how they perform the task.

Response: Thank you for raising this concern. We have described the phrase more operationally as follows, "the monkeys reported a target location (e.g., *Top-left* in the trial n, Fig 1c), which corresponded to a location assigned to the item-cue (e.g., *co-location I*) on the tilted map image of the context-cue (e.g., *-90°*)", to avoid interpretation in the revised manuscript (Page 8, Lines 110-114).

Comment 4: Lines 109-110: Here or in Methods, please specify the degrees of visual angle (DVA) that surrounded each target spot, and the amount of time the monkey needed to dwell on the target to complete the choice.

Response: Thank you for bringing this point to our attention. The fixation window was typically less than 2° from the center. The amount of time that the monkey needed to dwell on the target to complete the choice was 200 ms. We have revised the manuscript according to your suggestion (Page 21, Line 393 - Page 22, Line 395).

Comment 5: Lines 143-144: the authors suggest the co-location effect is due to item-item associative memory (mediated via the map). Can the authors rule out the alternative possibility that —during the item-cue period and ensuing delay—the neurons are doing prospective coding of the saccade and/or target location on the screen? Wouldnt that also lead to a co -location effect?

Response: We suggest that the co-location effect is due to the item-location associative memory. Yes, prospective coding of the saccade and/or target location on the screen also requires item-location associative memory and can lead to a co-location effect. We investigated this possibility as shown in original Figs 6-7 (current Figs 5-6). The advantage of examining the co-location effect is that we can find item-location associative memory even though neurons do not perform such prospective coding.

Comment 6: Lines 157-159: this sounds like cherry picking. What is the rationale for analyzing only neurons with high co-location indices? Fig. S4 aside, what happens if you analyze all neurons with significant co-location effects?

Response: Thank you for pointing it out. Due to the small number of pairs (n = 4), the corresponding significant value becomes extremely high ($r > 0.95$, $d.f. = 2$, $P < 0.05$, two-tailed). Therefore, we used the present threshold (r > 0.8). We know that it is arbitrary. Therefore, we showed the result with another threshold (r > 0.7) in the Supporting information to show that the present result did not necessarily depend on a particular threshold value. We also analyzed it using all neurons with significant co-location effects and presented it in Supporting information (Fig S4b). It showed the same tendency as the results with thresholds r > 0.8 and r > 0.7.

Comment 7: Lines 226-231: the authors find a significant relationship between coding for co-location during the item-cue period and coding for target location during the choice-fixation period (in the hippocampus and parahippocampal cortex) only when the map is oriented in the upright position (0 degrees). They then explain this may be due to a training effect. Doesnt this presen t a problem with the view that this is a mechanism for semantic-like retrieval? If that were the case, wouldnt there be significant relationships for more map orientations?

Response: Thank you for raising this point. Our previous study using the same ILA task (Yang and Naya, 2020) showed that coding for co-locations was fitted to the map of the background-cue (i.e., context-cue) in the HPC. In short, even though the co-location was represented relative to an upright map in the HPC, this self-referenced location can be subsequently fitted to the current spatial context given by the context-

cue, not only when the context-cue is 0° (upright). Thus, the retrieved co-location reflects an allocentric coding that can be used in multiple contexts, rather than a fixed-form association in only one context. Altogether, we do not think it is a problem that coding for co-location during the item-cue period had a significant relationship with coding for target location during the choice-fixation period. We have added these points in Discussion section of the revised manuscript (Page 18, Line 331 - Page 19, Line 339).

Reference:

Buzsáki, G., McKenzie, S., and Davachi, L. (2022). Neurophysiology of Remembering. Annual review of psychology 73, 187-215.

Higuchi, S., and Miyashita, Y. (1996). Formation of mnemonic neuronal responses to visual paired associates in inferotemporal cortex is impaired by perirhinal and entorhinal lesions. Proceedings of the National Academy of Sciences of the United States of America 93, 739-743.

Insausti, R., Annese, J., Amaral, D.G., and Squire, L.R. (2013). Human amnesia and the medial temporal lobe illuminated by neuropsychological and neurohistological findings for patient E.P. Proceedings of the National Academy of Sciences 110, E1953-E1962.

Levy, D.A., Bayley, P.J., and Squire, L.R. (2004). The anatomy of semantic knowledge: Medial vs. lateral temporal lobe. Proceedings of the National Academy of Sciences 101, 6710-6715.

Murray, E.A., Wise, S.P., and Graham, K.S. (2017). The evolution of memory systems: ancestors, anatomy, and adaptations (Oxford, UK: Oxford University Press).

Naya, Y., Yoshida, M., and Miyashita, Y. (2001). Backward spreading of memory-retrieval signal in the primate temporal cortex. Science 291, 661-664.

Tyler, L.K., Chiu, S., Zhuang, J., Randall, B., Devereux, B.J., Wright, P., Clarke, A., and Taylor, K.I. (2013). Objects and Categories: Feature Statistics and Object Processing in the Ventral Stream. Journal of cognitive neuroscience 25, 1723-1735.

Yang, C., and Naya, Y. (2020). Hippocampal cells integrate past memory and present perception for the future. PLoS biology 18, e3000876.

Responses to Reviewer #2:

Thank you for the recognition of our work and for the important suggestions and comments on the description of our manuscript. We have addressed all the comments listed point-by-point and have revised our manuscript accordingly.

Comment 1: In Fig.4a, the authors showed that the co-location index was significantly high in HPC, PRC, and PHC, but not in TE. The result would become more solid if the data from individual monkeys are also shown separately.

Response: Thank you for this suggestion. We showed the co-location index separately for each monkey in a new Fig 4b in the revised manuscript. We presented the data in cumulative frequency histograms to compare the area-difference patterns in co-location index between the two monkeys, while we kept the original figure presenting the co-location index from the two monkeys in a standard distribution histogram (Fig 4a in the revised manuscript) to give a more direct impression of the results.

Comment 2: In Fig.4b, the authors demonstrated that the population-averaged co-location indices over time from the item-cue onset have increased earlier in the PRC than in the HPC and PHC in the selected neurons with high co-location indices ($r > 0.8$). However, I wondered whether this analysis with the population-averaged co-location index may have masked the presence of a small population of early response-onset neurons in the HPC and PHC. Since this is one of the core observations in this

study, it would be better if an index from individual neurons supports the same conclusion. For example, the time of onset when the co-location index deviates significantly from the baseline should be determined in each neuron, and the distribution of the time of onset in each MTL area should be further analyzed and compared.

Response: Thank you very much for giving us the opportunity to reconsider this problem. We examined only the population-averaged time course of the co-location index in the original manuscript because a small pair number (n=4) of the co-location index consequently elicited a large noise in single-neuron-based statistics. For example, a significance at $P < 0.05$ requires $r > 0.95$ ($d.f. = 2$, two-tailed), which means that each neuron may easily show a large value of the co-location index value by chance. This problem is crucial particularly when we examine a time course of the co-location index because it contains many time points for the index (i.e., multiple-comparison effect). Some neurons showed large values of the co-location index even before the item-cue presentation (Fig S5a) although we expect the co-location index to be zero value. Therefore, the population-averaged time course is useful not only for depicting the overall characteristics of each area but also for canceling the unavoidable noise according to the central limit theorem.

However, we agree that it is important to analyze the time course at the single-neuron level. We have addressed this problem in the revised manuscript by calculating the co-location index on a single-trial base and averaging the time course for each neuron, which eliminated the noise by maintaining the signal (refer to Methods

section for details; Page 26, Line 491 - Page 27, Line 511; Fig S5). We determined the latencies in elevations of the index for individual neurons and confirmed the present result that PRC neurons increased the index earlier compared to HPC neurons. We have added a new figure (Fig 4d) and descriptions in the revised manuscript (Page 10, Lines 167 - Page 11, Line 170).

Comment 3: In the present study, the authors analyzed the "first-person perspective effect of the retrieved location" during the choice-fixation period. However, they performed the same analysis during the "background-cue period" (i.e., Background-cue period plus Delay 2 period) with HPC neurons in their previous study (Yang and Naya, 2020). It is interesting to see the results of the same analysis of the "background-cue period" data from each MTL area (i.e., not only HPC but also PRC and PHC) just as done in their previous paper, because more neurons would have shown task-related activity in both the item-cue period and background-cue period in the PRC and PHC, than in both the item-cue period and choice-fixation period. Specifically, the representation of task-related information during the "background-cue period" should be examined by applying a three-way (item-cue, context-cue, and target) ANOVA for each neuron in each MTL area and should be displayed just as for the choice-fixation period data in the S1 table and Fig.5 of the present paper. It is interesting to examine the "Similarity of orientation tuning" and the "Matching index" in the "background-cue period" and display the results for each MTL area, as shown in Fig.4c and Fig.5c of the previous paper. The results of the RSA may also be

displayed for the data from the "background-cue period," just as for those from the choice-fixation period shown in Fig.7b of the present paper.

Response: Thank you for bringing this point to our attention. This study focused on the item-cue period to investigate the recall process in the absence of spatial context. The choice-fixation period was utilized for this purpose but not to investigate itself. We apologize that the original manuscript might be misleading and that the reviewer might have misunderstood it as “the authors analyzed the ‘first-person perspective effect of the retrieved location’ during the choice-fixation period.” To clarify the role of the choice-fixation period in this study, we moved the original Fig 5 to the Supplementary information (Fig S6) because it is not directly related to the main goal of this study.

We chose the choice-fixation period for the comparison because we could collect neural responses related to relatively simple sensory-motor signals representing the external space where the monkeys gazed during that period. In contrast to the choice-fixation period, the cognitive demand during the background-cue (context-cue) period was more complex. We are grateful that the reviewer is interested in the results during the background-cue period. However, because they are not related to the main goals of this study, we believe it would be more appropriate to present them in a separate paper.

Minor comments

Comment 1: The authors used the term, "semantic-like memory" (rather than "semantic memory"), in the full title of this paper. However, they neither provided any discussion nor cited any references for their choice of this term. Please discuss this issue and provide appropriate references.

Response: Thank you for raising this issue. We used the term, “semantic-like memory,” because “semantic memory” was originally defined in the previous literature investigating human memory and it is still controversial whether non-human animals have semantic memory or not (Murray et al., 2017; Suddendorf, 2013). We added descriptions related with this point (Page 5, Line 75 – Page 6, Line 81).

Comment 2: P.12, lines 226-229: The authors suggest that the selective conjunction of the co-locations and the 0° map image might be due to the training history in which the monkeys had learned the item-location association under the context-cue with 0° orientation during the initial training ("default condition"). Do the authors have any independent evidence to support this suggestion?

Response: Thank you for your question. We are sorry we do not have independent evidence or reference to support this notion.

Comment 3: P.5, line 90: some typos?

Response: Thank you for highlighting this issue. We have revised the sentence, including line 90, to make it clear for readers in the revised manuscript (Page 6, Lines 92-95).

Responses to Reviewer #3:

Thank you for the recognition of our work and for the important suggestions and comments on the description of our manuscript. We have addressed all the comments listed point-by-point and have revised our manuscript accordingly.

Comment 1: My main concern resides on whether the process studied in the present paper is truly semantic or semantic-like memory recall for item-location pairs and not a more episodic form of this memory which could also be recalled by the two strategies suggested (allocenric versus first person perspective). How can author rule out this is not the case? To my opinion, this is not yet convincing enough (but might become upon further clarifications and discussion of the topic). Since this point has obviously a major impact on the interpretation of the data, it should be thoroughly addressed and substantiated.

Response: Thank you for highlighting this point. Accordingly, we have discussed whether the item-location associative memory in this study is semantic-like or episodic-like in the revised manuscript (Page 19, Lines 339-355). In a trial of the ILA task, the item-cue was followed by the context-cue, choice, and reward delivery and was not directly related to its co-location during the item-cue period. Episodic memory should contain these sequences of task events with a particular spatiotemporal context, including preceding and following trials, which are not necessary for task performance. It seems unlikely that the monkeys repeated remembering an episode of a particular trial during the item-cue period in each of the

more than 500 trials in every recording session, although we cannot deny that the monkeys might remember episodic-like memory in some trials.

Comment 2: As a side point, in 'the Retrieval of item-location associative memory p9, Fig 4b' part: the main results is that the co-location index increases earlier in the PRC than the HPC and PHC. Statistics supporting this claim should be included in the results section. In addition, the co-location index appears to also increase earlier in the HIP than the PHC, which is not especially expected. Please provide also these comparisons and further extend the discussion on the role of the PHC within this framework.

Response: Thank you for raising this issue. According to the reviewer, we have added the statistics of the original Fig 4b (current Fig 4c) to the main text for both comparisons in the revised manuscript (Page 10, Lines 159-164). We also extended the discussion on the role of the PHC as a carrier of the retrieved location from the HPC to the parietal cortex in the dorsal stream (Page 18, Lines 320-330).

Comment 3: Also, as much as I have sympathy for the limited space allocated in abstracts, a statement such as the standard consolidation theory suggests....while the PRC is involved in its long-term storage (i.e. semantic memory)" ...shall definitively be rephrased as to my knowledge the SCT has not made such a specific claim

especially involving the PRC and/ or its involvement in semantic memory, nor did the senior author of the present manuscript in a previous study (see Naya et al,

2016: The perirhinal cortex represents both between-domain associations (e.g., item-reward, item-place and item-time) and within-domain associations (e.g., item-item) and contributes to both subcategories of declarative memory (i.e., episodic and semantic memory) in a way that is complementary with the hippocampus".

Response: Thank you for highlighting this point. We agree that we have specified the theory too much. Accordingly, we have revised the text in the revised manuscript (Page 2, Lines 13-15).

Comment 4: - some discussion parts have found their way to the results sections (example p9)- they probably should not; Figures: Fig 4a and b) add names of the brain areas directly on the graph, legends: spell out the message to take home in each legend, run a spell check for typos.

Response: Thank you for raising this point. According to the reviewer's suggestion, we reduced descriptions about the relationship between the present results and previous studies in the result section in the revised manuscript (Page 11, Lines 172-174). We have also revised Fig 4 and the legends accordingly.
