# Supplementary material for: Sequential involvements of the perirhinal cortex and hippocampus in the recall of item-location associative memory in macaques
Source: PLoS Biol. 2023 Jun 8;21(6):e3002145. doi: 10.1371/journal.pbio.3002145 (PMC10284415; doi:10.1371/journal.pbio.3002145)
Supplement: Attachment — Submitted filename: Response_Yang_Naya.pdf [file pbio.3002145.s014.pdf]

Responses to Reviewer #1:

Thank you for the recognition of our work and the important suggestions and comments on the description of our manuscript. We have addressed all the comments listed point-by-point and have revised our manuscript accordingly.

Comment 1: Line 66: the PHC is usually considered to reside in the ventral pathway. In the Discussion (e.g., line 254), the authors explicitly refer to the PHC as being part of the MTL. The authors should consider rephrasing this - perhaps something like PHC is a ventral stream structure that is also well connected with the dorsal pathway?

Response: We specified the connection pattern of the PHC according to your suggestion in the introduction of the revised manuscript (Page 5, Lines 65-70). In addition, we avoided referring to the PHC explicitly as being part of the MTL in the discussion of the revise manuscript (lines 260, 262, 263, 331). We did not rephrase it as a ventral stream structure because it is more common to consider the PHC as the MTL in memory research field (Squire, Stark Clark, 2004; Eichenbaum, Yonelinas, Ranganath, 2007) although we agree that the PHC can be also considered as a ventral stream structure particularly when we focus on visual processing.

Comment 2: Lines 154-156: the authors need to back-off this statement regarding correlation with of the physiological findings with behavioral effects of lesions. There are many contrary findings in both humans and monkeys regarding participation of

PRC in visual perception. Either remove the statement (starting with 'which is inconsistent') or acknowledge the contrary findings.

Response: You are right. The PRC is also involved in visual perception. We meant more preferential involvements of TE in visual object compared with the PRC in this sentence. We clarified this point in the revised manuscript (Page 10, Lines 158-159)

Comment 3: Lines 336-338: the sentence starting with 'Notably' is difficult to understand. Please recast or add explanation.

Response: Thank you for your suggestion. We reword it with 'However' in the revised manuscript (Page 19, Line 351).

Comment 4: Lines 338-354: I gather that the authors are trying to address the referee 3's comment that the task (and therefore the physiological correlates) may tax episodic memory. Isn't there a simpler explanation? Clearly the monkeys have been trained for thousands of trials with a particular small set of objects and the one map. This argues against the idea that the learning and recall expressed here by the monkeys is episodic-like. If the authors (and editor) agree, I suggest they replace the new text with the simpler explanation.

Response: According to your suggestion, we added the simpler explanation (Page 19, Lines 356-358). We actually thought in the same way as you suggested. However, we

kept the “new text” in the current revised manuscript because referee 3 suggested the possibility that the monkeys employed episodic memory in the ILA task although she or he also knows that “the monkeys have been trained for thousands of trials with a particular small set of objects and the one map” and we can’t reject that possibility completely only by the simpler explanation.

Comment 5:.Line 371: it is not very helpful to refer to a manuscript 'in press' for supporting evidence. If the information in the cited ms. is providing essential evidence for this statement/conclusion, it should be explained. Alternatively, if the data in the present paper is sufficient then the citation could be dropped.

Response: The manuscript was published. We corrected the information (Page 37, Lines 760-761).

Responses to Reviewer #2:

Thank you for the recognition of our work and for the important suggestions and comments on the description of our manuscript. We have addressed all the comments listed point-by-point and have revised our manuscript accordingly.

Major Comment: The authors' answer to my previous Major Comment 3 is not convincing enough for me. In response to my previous comment, the authors wrote 'We apologize that the original manuscript might be misleading and that the reviewer might have misunderstood it as "the authors analyzed the 'first-person perspective effect of the retrieved location' during the choice-fixation period".' However, in the authors' revised manuscript, the phrase 'first-person perspective' appears six times in the main text and once in the abstract. Even a section titled 'First-person perspective of the retrieved location' is included in the Results chapter. Therefore, a proper analysis of the 'first-person perspective effect' is essential in this paper to ensure the validity of the data, analysis and interpretation of the study, whether it is based on 'choice-fixation period' data or 'item-cue period' data.

I request the authors give a more detailed response to the previous Major Comment 3 by revising it again. In this paper, they must disclose results of the analysis of the "background-cue period" that I requested in my previous Major Comment 3 (probably in Supplementary Information). Disclosure of an analysis that includes all or part of the analytical methods I suggested in my previous Major Comment 3 would be preferable.

Response: The authors analyzed the “first-person perspective effect of the retrieved location” during the item-cue period in the present manuscript. This is one of the main points of this study. Therefore, we used the phrase “first-person perspective” several times in the manuscript including the abstract and the section title. We are afraid that the reviewer misunderstood as “In the present study, the authors analyzed the 'first-person perspective effect of the retrieved location' during the choice-fixation period” (Major Comment 3). We would like to apologize (again) for not explaining it well last time although we had made our best efforts.

“The proper analysis of the 'first-person perspective effect'” on neural activities during the item-cue period is to compare them with those during the choice-fixation period. We used neural activities during the choice-fixation as a reference of the “first-person perspective effect” because “we could collect neural responses related to relatively simple sensory-motor signals representing the external space where the monkeys gazed during that period” (*from the response to Comment 3*).

Before the comparison, we conducted the three-way ANOVA and confirmed that the PRC, PHC and HPC exhibited selective activity exclusively to target locations where the animals gazed during the choice-fixation period (Fig. S6a). This is only for confirmation and is not a goal of this study. In contrast to the activity during the choice-fixation period, our previous study reported the HPC exhibited selective activity to item-cue and context-cue substantially as well as target locations. Here, we show you the results of three-way ANOVA including the PRC and PHC that you primarily requested in Comment 3. Overall, all three areas signaled other task-related

information such as item and/or context-cues in addition to target locations. Because of the intermingled task-related signals, it would not be appropriate or difficult to use the activity during the context-cue period as a reference particularly when we apply population coding analysis such as cell-based RSA. In addition, it should be noted that the monkeys fixated at the center of the display during the context-cue period, which means that the activity during the choice-fixation period would be more reliable as a reference of the “first-person perspective effect”. We have added this rationale of the current analysis in the revised manuscript (Page 17, lines 298-306).

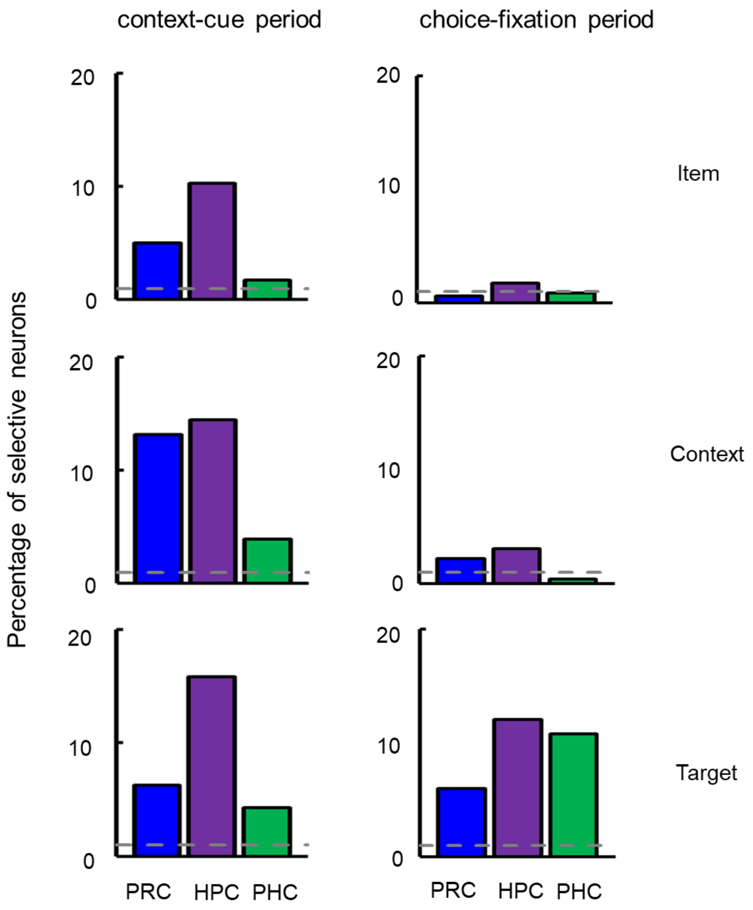

Task-related signals in MTL areas during the context-cue (left column) and choice-fixation (right column) periods. Percentages of item-cue, context-cue, and target-selective neurons out of the recorded neurons (three-way ANOVA). Dashed line, 1.0% chance level. PRC, perirhinal cortex (n = 319). HPC, hippocampus (n = 456). PHC, parahippocampal cortex (n = 232).

To examine the “first-person perspective effect” on neural activities during the context-cue (background-cue) period, it would be more appropriate to compare them with those during the choice-period than to conduct the same analyses as in our previous study. In any case, the results during the context-cue (background-cue) period would not substantially contribute to the present study, because our present goal is to examine recall process during the item-cue period.

We appreciate the reviewer for your interest in the results during the context-cue (background-cue) period. However, as shown in our previous study (Yang and Naya. 2020), neural process during the context-cue (background-cue) period was very complex and related with other topic concerning on constructive process to combine retrieved memory from the item-cue and incoming perception presented by the context-cue. We believe it is more appropriate to present the results in a separate paper rather than just to disclose them in this paper.

Minor Comment: In response to my previous Minor Comment 2, the authors replied 'We are sorry we do not have independent evidence'. This point is important and, besides me, Reviewer #1 also expressed her/his concern on this point in her/his Comment 7. The authors have added a new discussion in this revision (Page 18, Line 331-Page 19, Line 339). However, it would be preferable to further discuss possible explanations for this phenomenon other than 'training history'.

Response: We added another possible explanation in the revised text (Page 14, Lines 240-242).

Response to Reviewer #3:

Thank you for the recognition of our work and for the comment on the description of our manuscript.

Comment: "The authors have addressed all my concerns. I would recommend to remove the word 'Conversely' from the abstract as the first and second sentences do refer to content comparable in nature."

Response: We removed the word 'Conversely' in the revised manuscript (Page 2, Line 15).
