# Supplementary material for: Sequential involvements of the perirhinal cortex and hippocampus in the recall of item-location associative memory in macaques
Source: PLoS Biol. 2023 Jun 8;21(6):e3002145. doi: 10.1371/journal.pbio.3002145 (PMC10284415; doi:10.1371/journal.pbio.3002145)
Supplement: Attachment — Submitted filename: Response_Yang_Naya.pdf [file pbio.3002145.s015.pdf]

Responses to Reviewer #2:

Thank you for the important suggestions on the description of our manuscript. We appreciate your devotion to the reviewing process of our manuscript. We have addressed all the comments listed point-by-point and have revised our manuscript accordingly.

Comment 1: The figure presented in "Response to Reviewer #2" (Figure X) is informative. This figure displays the results of a three-way ANOVA, including the PRC, HPC, and PHC, during the context-cue period. It is slightly different from the one I requested (i.e., I requested the data during "Context-cue plus Delay-2 period," because the "item-cue period" consists of "item-cue plus Delay-1 period" according to the authors' definition). The figure (Figure X) should be shown in Supporting Information (a partial overlap with Figure S6a needs to be adjusted).

Response: According to the reviewer's suggestion, we showed the figure (Figure X10) in Supporting Information (Fig. S10). The "Figure X" displays results during "Context-cue plus Delay-2 period." The "context-cue period" consisted of "context-cue plus Delay-2 period" as we defined the item-cue period as "item-cue plus Delay-1 period" in the main text. We noted this definition of "context-cue period" explicitly in a legend of Fig. S10.

Comment 2: Figure X would be more informative if the results of the three-way ANOVA during the item-cue period were included in the figure in the same format as the results during the context-cue period (Expanded Figure X). Although some of the ANOVA results for "Item" are already shown in Figure 2b and Table S1, full disclosure of the ANOVA results including the "Context" and "Target" factors will help the reader understand the signal content of the PRC, HPC, and PHC neurons during each phase of this task. I suggest including that figure (Expanded Figure X) in Supporting Information (the overlap with Figure 2b and Table S1 can remain unadjusted).

Response: According to the reviewer's suggestion, we showed the results of the three-way ANOVA during the item-cue period in Supporting Information (Fig. S10).

Comment 3: As I noted in my previous comments on the R2 manuscript, I believe that "proper analysis of 'first-person perspective effects' is essential in this paper to ensure the validity of the data, analysis, and interpretation of the study, whether it is based on 'choice-fixation period' data or 'item-cue period' data." With the results during the context-cue period shown in Figure X, I take the same position. I believe that an appropriate analysis of the context-cue period data is important, including all or some of the analytical methods that I had suggested in my Major Comment 3 on the R1 manuscript (Figure X shows that it is possible). It is favorable that the results of such

analyses be disclosed in this paper, but if the authors plan to present those results in another paper, I look forward to reading it.

Response: The present study focused on retrieval of the item-location association memory in the absence of a particular context information. For this purpose, we examined 'first-person perspective effects' on neural representation during the item-cue period (starting from the item-cue presentation and before the context-cue presentation) by comparing it with neural representation during the choice-fixation period. As we noted in our previous responses to reviewer's comments, this would be "the proper analysis of the 'first-person perspective effects' on neural activities during the item-cue period at least in the present study because it is difficult to examine the 'first-person perspective effects' on neural activities during each task period by themselves. Unfortunately, the present task paradigm did not require the animals to report their subjective mental image at each time point. It is a limitation of the present study. We have discussed this point in the revised manuscript (Page 17, Lines 314-317). Referring to the new Fig. S10, we confirmed the rationale to use the choice-fixation period rather than the context-cue period for the analysis to examine the 'first-person perspective effects' during the item-cue period in the revised manuscript (Page 17, Lines 303-314).
